# Double-blind placebo-controlled randomized clinical trial on the use of paracetamol for performing mammography

**DOI:** 10.1097/MD.0000000000010261

**Published:** 2018-03-30

**Authors:** Ruffo Freitas-Junior, Edesio Martins, Cristiane Metran-Nascente, Angela Assis Carvalho, Marilceia Ferreira da Silva, Leonardo Ribeiro Soares, Carlos Alberto Ximenes

**Affiliations:** a Program of Mastology, Federal University of Goias; b Arthesanal Pharmacia; c Department of Radiology, Federal University of Goias, Goiânia, GO, Brazil.

**Keywords:** breast, discomfort, mammography, pain, paracetamol

## Abstract

**Background:**

This study was conducted within the Goias Mastology Research Network. To verify the possibility of diminishing pain, and discomfort during the mammography using analgesic administration.

**Methods:**

Randomized, double-blinded, placebo controlled trial, testing paracetamol to diminish the pain, and discomfort during mammography. Three hundred patients who came for screening mammography were randomized for this study. A questionnaire with 2 parts was used: the first had questions that concerned the patient identification, and factors related to the pain during mammography; and the second asked about the scale of discomfort (no discomfort; uncomfortable; very uncomfortable; intolerable), and the pain (analogical linear scale) during the mammography. Each patient received 1000 mg of paracetamol, or placebo. Afterwards each patient filled out the second part of the questionnaire. Six patients were excluded from the analysis; this resulted in 149 in the paracetamol group, and 145 in the placebo group.

**Results:**

The 2 groups were homogenous concerning the mean of the ages, weight, height, and breast size. The mean of the pain was 3.5 in the paracetamol, and 2.8 in the placebo group (*P* = .12). There were fewer women experiencing mild pain in the paracetamol group when compared with those in placebo group (relative risk [RR] 0.76, confidence interval [CI] 95% 0.52–0.98). There was no significant difference between the 2 groups, according to the degrees of discomfort (*P* =  .69).

**Conclusion:**

The use of paracetamol can reduces the mild pain for women undergoing mammography.

## 1 Introduction

Mammography has been used worldwide in breast cancer screening programs.^[1,2]^ The screening mammogram can result in earlier diagnosis, and thus identification, and treatment of smaller size tumors. In previous studies, this practice was associated with more conservative treatments, and reduction of mortality due to breast cancer.^[1–3]^

However, mammographic examination frequently causes pain, and discomfort while being performed.^[4]^ These complaints result from compressing the breast so that the woman is exposed to radiation for as little time as possible, and to improve the image quality.^[5,6]^ It has also been determined that the pain during the examination may be related to various factors such as the time within the menstrual cycle, anxiety, caffeine use, and previous history of mastalgia.^[7–10]^

One of the possible interventions aimed at soothing any pain, and discomfort felt during mammographic examination is analgesia, which can be achieved by using paracetamol. This drug is indicated for symptomatic treatment of pain, and fever, and it is considered one of the safest analgesics.^[11]^ For this purpose, it acts predominantly, by inhibiting the synthesis of prostaglandins in the nervous system, and, to a lesser extent, by peripherally, blocking pain generation.

Thus, the present study was conducted with the aim of studying the possibility of diminishing the pain, and discomfort when performing mammography, by means of the use of paracetamol.

## 2 Patients and methods

### 2.1 Study design

**Figure 1:**
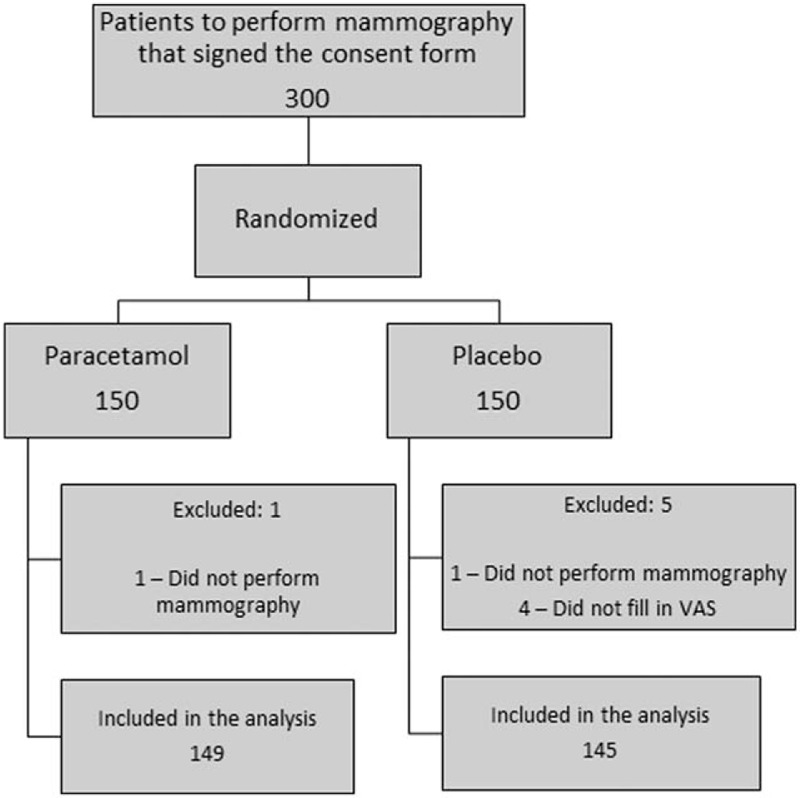
Study design, and distribution of patients into the groups. VAS = visual analog pain scale.

This was a double-blind placebo-controlled randomized clinical trial to test the use of paracetamol for diminishing the pain, and discomfort while performing mammography (Fig. 1).

### 2.2 Sample size

Considering that approximately, 55% of the patients in the placebo group would have pain during mammography; and expecting that a gain of 15% in the medication group, it would be necessary to have 134 patients in each branch of the study in order to observe any difference greater than 15%. Sample rejection of up to 10% was taken into consideration, along with exclusion criteria. Two-tailed analysis, with a power of 80%, and the *P* value set at .05 were predefined. Thus, 300 patients were included in the study, distributed randomly, between the 2 groups.

### 2.3 Inclusion criteria

Women with a prior indication for mammography who were over 40 years old were included. These patients would undergo their radiological examination at the university hospital of the Federal University of Goiás. Patients with any mammary skin lesions or with history of intolerance to paracetamol were not allowed in the study.

### 2.4 Entry into the study

Before performing the mammography, the patients were approached by one of the members of the study team, who gave detailed explanations about the study, its aim, and the possible benefits, and side effects. Each patient was signed a free, and informed consent statement form. The same member of the study team was then available for clarifying any possible queries that the patients might have. After that, the patient received an envelope containing 2 capsules of 500 mg of paracetamol (totaling 1000 mg), or 2 capsules of placebo.

### 2.5 Randomization

The randomization was performed by means of computer generation sequencing. Neither the investigators nor the patient knew how the group allocations were organized. The record book remained in the hands of the pharmacist of the study who, in turn, did not have any contact with the patients, or with the interviewer.

### 2.6 Preparation of the medication

The Farmácia Artesanal (Artesanal Pharmacy), a compounding pharmacy that is duly licensed and certified by the official regulatory agencies of the city of Goiânia (Goiás, Brazil), and that possesses the appropriate analytical, and quality assurance certificates, formulated the medications (placebo or paracetamol).

The pharmacist of the study, in the laboratory, was responsible for organizing the study medications by means of standard procedures, and quality control. The medications were prepared as follows: active group, with 500 mg of paracetamol per capsule for gastric degradation; and placebo group, with 350 mg of starch per capsule for gastric degradation.

The external appearance of the capsules for the 2 groups was identical. There was a sealed code, organized by the pharmacist responsible for the medications, and was not supplied to the other team members involved in the study. This remained sealed, unless there was the need to break the secrecy because of an allergic reaction presented by any of the patients.

### 2.7 Use of the medication

After each patient received the medication, she also received a glass of water. The patients took the medicine orally, with the water, 1 hour before the procedure.

### 2.8 Mammography procedures

The mammography at the university hospital was carried out using a high-resolution mammography machine with mean compression in each plane of approximately 800 newtons.

All the patients underwent examination using at least 4 imaging views: 1 craniocaudal, and 1 mediolateral oblique plane for each breast. When necessary, complementary imaging was used, such as with selective compression, magnification, or other methods.

### 2.9 Collection of information on pain and discomfort

After each patient had undergone the mammographic examination, she was again approached by the same member of the study team, who gave her a linear visual analog pain scale (VAS), and asked her to mark the intensity of the pain she experienced on this scale. The patient was also asked to describe her discomfort during the mammographic examination.

### 2.10 Control variables

- Patient's age in completed years at the time of the study;
- Menopausal status;
- Current use of hormone replacement therapy;
- Previous history of mastalgia;
- Daily coffee consumption;
- Bra size;

### 2.11 Dependent variables

The pain was measured according to the linear VAS. The degree of pain was classified then as mild pain (0.1–2.5); moderate pain (2.6–7.0), and a lot of pain (7.1–10).

Discomfort was classified into 4 possible categories according to the patient's opinion: no discomfort, uncomfortable, very uncomfortable, or intolerable.

### 2.12 Statistical analysis

The control variables were used to check the homogeneity of the groups, tested using Student's *t* test, or the Chi-square test, as applicable. The pain intensity according to the visual analog scale was tested according to the mean, using Chi-square test. The discomfort between the groups was tested using the Chi-square test. A multivariate logistic regression analysis was performed to determine the risks for pain, and discomfort during mammography. The nullity hypothesis was rejected if there was a difference with significance greater than 95% between the groups (*P* <  .05). The statistical analyses were performed by SPSS 18.0 (SPSS Inc., Chicago, IL) software, *MedCalc* for Windows, version 17.9 (*MedCalc* Software, Ostend, Belgium), and Forest Plot Generator (DistillerSR, Evidence Partners, Ottawa, CA).

### 2.13 Ethical matters

This study was approved by the Research Ethics Committee of the university hospital of the Federal University of Goiás (registration number 026/2002), and it was conducted in accordance with the ruling principles of the Helsinki Convention. The participants were volunteers, and they signed a free, and informed consent statement before their inclusion in the study, having received information about the study, its aim, and its probable side effects, and future advantages.

## 3 Results

Out of the 300 women included, 6 were excluded from the analysis: one did not undergo mammography because of technical problems with the equipment, another was unable to complete the examination because of the intensity of the pain experienced, and the other 4 did not fill out the questionnaire, despite having undergone mammography. Thus, 294 patients were analyzed. The paracetamol group consisted of 149 women, while the placebo group had 145 (Fig. 1). The randomization of the patients between the groups made it possible for the groups to be homogenous, such that all the control variables were statistically, similar between the two groups, including the factors that could possibly, interfere with the intensity of the pain while the mammography was being performed (Table 1).

**Table 1:**
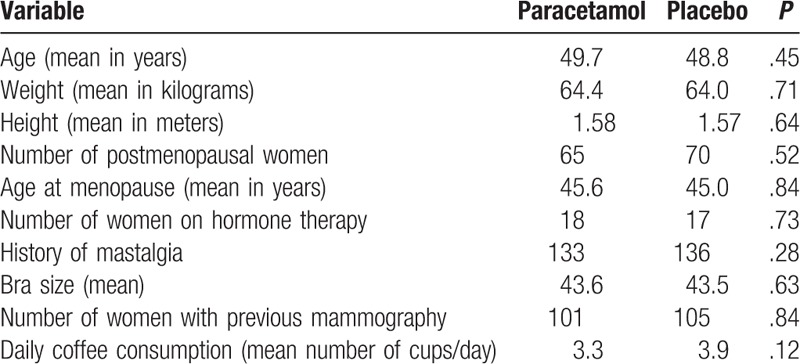
Comparison of the demographic variables between the 2 groups: paracetamol and placebo.

**Figure 2:**
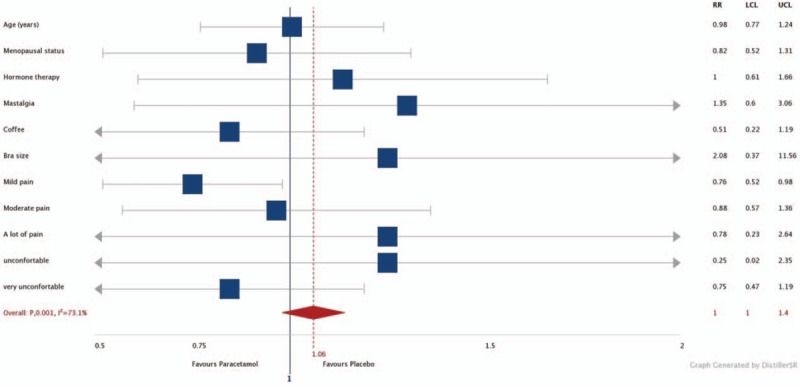
Forest plot for subgroup analysis.

The mean of pain experienced during the examination, according to the VAS, was 3.5 for the paracetamol group, and 2.9 for the placebo group (*P* =  .12). There were fewer women experiencing mild pain in the paracetamol group when compared with those in placebo group (relative risk [RR] 0.76, confidence interval [CI] 95% 0.52–0.98) (Table 2, Fig. 2). There was no significant difference between the two groups, according to the degrees of discomfort (Table 3).

**Table 2:**
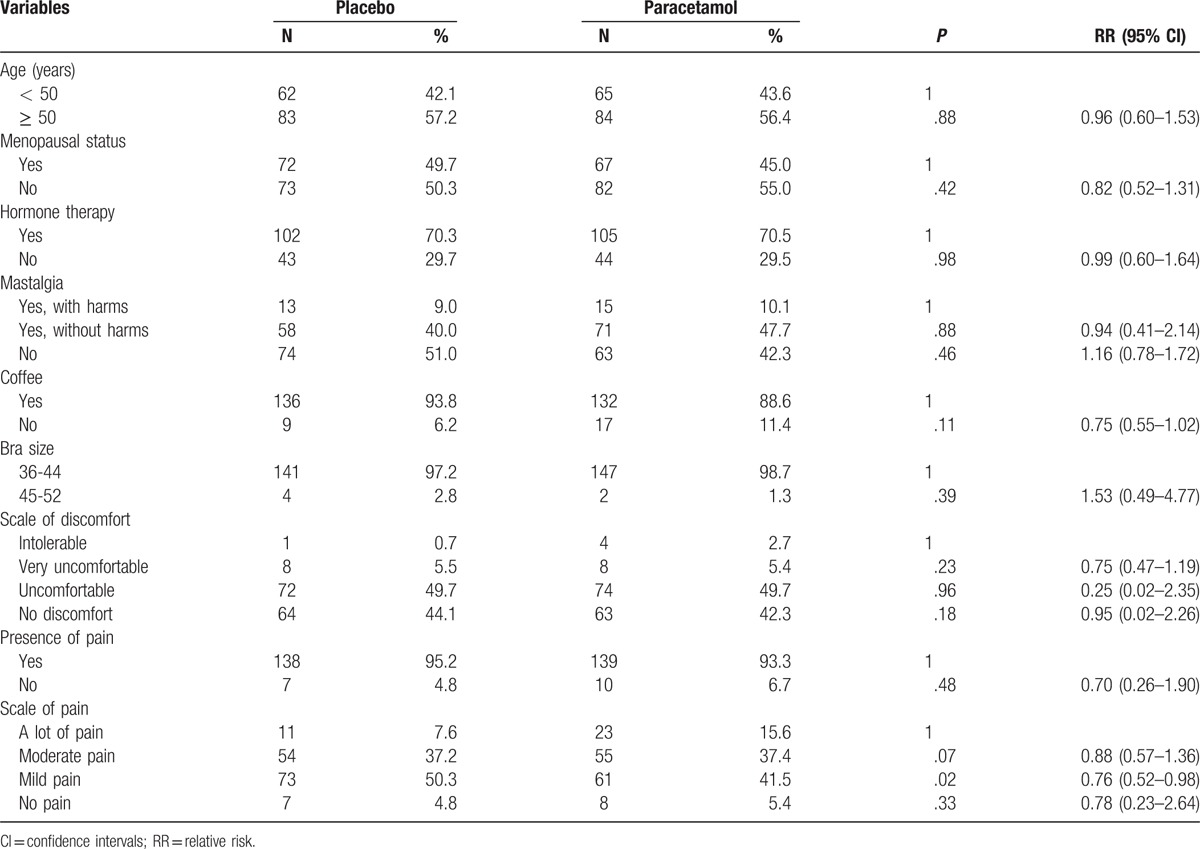
Multivariate analysis of the clinical variables by logistic regression.

**Table 3:**
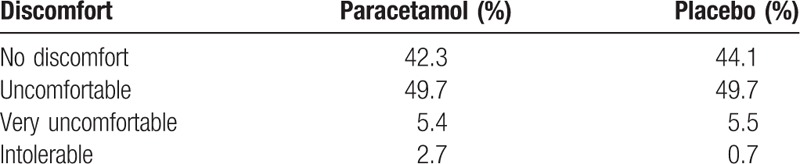
Degree of discomfort experienced by patients during mammography, according to the group (*P* =  .69).

Regarding the safety, except for one women who presented slight sleepiness after taking the medication (allocated to the paracetamol group), there were no other adverse events.

## 4 Discussion

The proportion of women who reported feeling pain during mammography varies widely.^[12–14]^ Up to 60% of these women can have a mild, or even a moderate discomfort.^[15]^ However, the reliability, and validity of these measurements are debatable. Some studies that found low frequencies of reports of pain were based on questions asked by the professional team at the time of performing the mammography.^[16,17]^ Other studies that also reported low frequencies of pain were based on investigations carried out sometime after the mammography was performed.^[18]^ Moreover, the amount of pain reported depends greatly, on the way in which the question is asked.^[16,18]^

It should be noted that the pain measurement can be performed by several quantitative questionnaires, and methods.^[16,17,19]^ In this context, despite some criticism as the non-linearity of the painful representation,^[19]^ the VAS remains the main tool in clinical studies.^[20,21]^

In the current study, there was no difference in mean score of VAS among the groups. However, the evaluation of pain intensity divided by subgroups revealed benefit of paracetamol in the reduction of mild pain during mammography. Thus, the use of paracetamol as premedication can benefit most women who reported pain during mammography, due to the prevalence of mild pain among those who reported pain symptoms.

In addition to the technical variations relating to the manner, and timing of asking the questions, there are also individual variations that cause some women to feel pain more than others do. These variations are generally evaluated in relation to demographic variables (age and education), or individuals’ medical histories (smoking, caffeine use, menstrual cycle, and menopause). However, these variables may not be consistently, associated with the degree of pain during mammography.^[10,13]^ In the current study, none of the analyzed factors significantly, associated with the frequency of pain, or discomfort during the exam. Still, this information can help to identify specific subgroups, which might have higher probability of experience pain, and discomfort, such as women with previous history of mastalgia.^[7]^ Such knowledge allows a customized patient approach avoiding breast discomfort, and pain.

Currently, despite the evolution of mammography equipment, and ongoing training of health professionals,^[22,23]^ the prevalence of breast discomfort remains frequent in mammography screening programs.^[4,24]^ In the current study, the presence of pain, or breast discomfort was observed in most women studied, which is in accordance with other similar studies.^[14,20]^ These data reinforce the need for strategies for the control of pain, and breast discomfort during mammography.

To the best of our knowledge, only 2 other attempts have been made to administer medication prior to mammography with the aim of reducing the pain, and discomfort. In one study, acetaminophen was used prior to the mammographic examination, and was compared with placebo, with no observed reduction in breast discomfort among women who used the medication.^[21]^ In another study, women were randomized to use paracetamol, ibuprofen, and/or lidocaine gel. Only premedication with lidocaine gel significantly, reduced discomfort during mammography.^[20]^

Among the limitations of the study, it should be emphasized the subjective measurement of breast discomfort, considering the absence of validated scales for this evaluation. The quality of the mammography in the 2 groups was not evaluated because the medication did not interfere in the image acquisition process.

We are now trying out new alternatives for medications to reduce the complaints relating to mammography. The possibility of using agents with greater analgesic power may be a good strategy for reducing this problem that affects millions of women worldwide. Considering the safe profile, and clinical benefit in the evaluation by subgroups, paracetamol can be used as a premedication before mammography, and would be included in new clinical research trials for the control of pain, and breast discomfort.

## 5 Conclusion

The use of paracetamol before performing mammography reduces the mild pain caused by the radiological examination, and combined with its safe profile should be used as a premedication before mammography. The study design, and the homogeneity obtained validate the results, although new clinical trials should be conducted with the aim of seeking a solution for women that experience more intense discomfort, and pain during mammography.

## Author contributions

**Conceptualization:** A.A. Carvalho, C.A. Ximenes, C. Metran-Nascente, M.F. Silva, R. Freitas-Junior.

**Data curation:** A.A. Carvalho, C. Metran-Nascente, M.F. Silva.

**Formal analysis:** C.A. Ximenes, E. Martins, L.R. Soares.

**Investigation:** A.A. Carvalho, C. Metran-Nascente, E. Martins, M.F. Silva.

**Methodology:** A.A. Carvalho, E. Martins, L.R. Soares, M.F. Silva, R. Freitas-Junior.

**Project administration:** C.A. Ximenes.

**Resources:** A.A. Carvalho, C. Metran-Nascente.

**Software:** E. Martins.

**Supervision:** C.A. Ximenes, R. Freitas-Junior.

**Validation:** L.R. Soares.

**Visualization:** L.R. Soares.

**Writing – original draft:** A.A. Carvalho, C.A. Ximenes, C. Metran-Nascente, E. Martins, L.R. Soares, M.F. Silva, R. Freitas-Junior.

**Writing – review & editing:** A.A. Carvalho, C.A. Ximenes, C. Metran-Nascente, E. Martins, L.R. Soares, M.F. Silva, R. Freitas-Junior.
